# Normal Hematopoetic Stem and Progenitor Cells Can Exhibit Metabolic Flexibility Similar to Cancer Cells

**DOI:** 10.3389/fonc.2020.00713

**Published:** 2020-05-12

**Authors:** Marija Vlaski-Lafarge, Veronique Labat, Alexandra Brandy, Alice Refeyton, Pascale Duchez, Laura Rodriguez, Nyere Gibson, Philippe Brunet de la Grange, Zoran Ivanovic

**Affiliations:** ^1^R&D Department, Etablissement Français du Sang Nouvelle Aquitaine, Bordeaux, France; ^2^Inserm/U1035, University of Bordeaux, Bordeaux, France

**Keywords:** cancer stem cells, hematopoietic stem cells, metabolism, bioenergetics, mitochondrial respiration

## Abstract

It is known that cancer stem cells (CSCs) with the largest proliferative capacity survive the anoxic and/or ischemic conditions present inside tumorous tissue. In this study we test whether normal stem cells can survive under the same conditions due to cancer cell-like metabolic adaptations. We cultivated a CD34^+^ population with a majority of hematopoietic progenitors, and a CD34^+^CD38^low^CD133^+^CD90^+^CD45RA^−^ population, highly enriched in hematopoietic stem cells (HSCs), under anoxic, anoxic/aglycemic (“ischemia-like”), or physiological conditions (3% O_2_). Results showed, despite a reduction in total cell fold expansion proportionate to the decrease in O_2_ concentration; CD34^+^ cells, aldehyde dehydrogenase-expressing primitive cells, and committed progenitors expanded, even in anoxia. Interestingly, under ischemia-like conditions, stem and CD34^+^ cell populations are maintained at day-0 level. Cell-cycle analysis further revealed an accumulation of cells in the G0/G1 phase in anoxia or anoxia/aglycemia, with a fraction of cells (~40%) actively cycling (SG2M phases). Also stem cell analysis showed that in these conditions a long-term Scid Repopulating activity was equal to that found with 3% O_2_. In addition stem cells with the highest proliferative capacity were maintained in anoxia/aglycemia and in anoxia. The estimated ATP profile, active mitochondrial content, and succinate accumulation are indicative of anaerobic mitochondrial respiration in both HSCs and CD34^+^ progenitors under ischemia-like conditions. We demonstrate here that primitive hematopoietic cells show similar metabolic flexibility to CSCs, allowing them to survive a lack of O_2_ and O_2_/glucose. Our study reveals that this feature is not the consequence of malignant transformation, but an attribute of stemness.

## Introduction

The concept of cancer stem cells (CSCs) is well-established (1). CSCs display functions similar to normal stem cells, including self-renewal, differentiation potential, migration, a high proportion of cells in G0 phase, and drug resistance. Also, as normal stem cells, CSCs represent the small fraction of tumor cell population capable of self-regeneration as well as of propagation of the malignant cell

population giving rise to new tumors in tissues other than those from which the cancer originates (2).

In addition, functional, normal, and CSCs share several metabolic traits. Cancer cells show flexible, metabolic reprograming phenotypes which use an array of energetic fuels to enable survival, and proliferation under various conditions (3, 4). Proliferating tumor cells tend toward a glycolytic metabolism, even under aerobic conditions, in order to acquire the precursors necessary for increased biosynthesis (5). This high demand for glycolysis occurs without loss of mitochondrial activity (TCA cycle/respiration) (6). It should be stressed that, contrary to common assumption, the Warburg effect is not in fact specific to malignancy (2). A glycolytic metabolic profile has been shown to be an intrinsic property of hematopoietic stem cells (HSCs), related to stemness, enabling the cells to reside in areas with extremely low O_2_ (hematopoietic niche). But this metabolic profile is also maintained when HSCs are outside of the physiological niche (2). Indeed, HSCs from bone marrow or mobilized in peripheral blood exhibit a glycolytic metabolic profile even when cultured under non-physiologically high atmospheric air O_2_ concentrations (7, 8).

However, both HSCs and cancer cells possess active mitochondria that contribute to their maintenance, and retain the ability for oxidative phosphorylation (6, 9). Besides their role in energy production, mitochondria also play a role in recycling NAD^+^ and maintaining the glycolytic flow, generating intermediates for anabolic pathways, epigenetic control, and protection against oxidative stress. They also have the ability to change swiftly to OXPHOS in order to meet the high energy demands linked to differentiation toward mature somatic cells (10).

In cancer cells, targeted depletion of mitochondrial DNA reduces the tumorigenic potential of cancer cells *in vitro* and *in vivo* (11, 12). Also, quiescent and circulating cancer cells rely highly on mitochondrial respiration (11, 13). The tumorous cells' metabolic flexibility between a predominantly biosynthetic or bioenergetic purpose is a result of this apparent dichotomy (glycolysis/mitochondrial respiration). Recent data show that the cancer cells with the greatest stem cell potential are responsible for the durability of the disease and can survive under severe conditions, such as anoxia and/or ischemia, created inside the tumor tissue. This ability to survive also depends on the metabolic consequences of anaerobic mitochondrial respiration. The mechanism described includes the use of fumarate as the final electron acceptor (fumarate respiration or “disproportionation of malate”) (14).

We thus want to test the hypothesis that HSCs, unlike mature cells, can survive under extreme conditions (anoxia and ischemia-like) due to metabolic adaptation, including anaerobic mitochondrial activity.

Our study, based on functional and metabolic analysis of HSCs, points to flexible energetic nature and high metabolic adaptability as being features common to stem cells, rather than specific to CSCs.

## Materials and Methods

### Cell Sorting and Culture

#### CD34^+^ Cell Isolation

Cord blood (CB) samples delivered (with the mother's approval) to the Cell Therapy Unit of the French Blood Institute, Bordeaux, that had been rejected for banking, were used for the experiments (In compliance with national French regulation, declared to the Ministry of Research: DC-2019-3720). CB CD34^+^ cells were isolated using an immunomagnetic technique (Miltenyi Biotec, Paris, France) and stored at −80°C (15).

#### CD34^+^CD38^low^CD133^+^CD90^+^CD45RA^−^ Cell Sorting

CD34^+^ cells were thawed in 4% human serum albumin (Vialebex, LFB-biomedicament, Courtabeuf, France) and labeled with anti-CD34-BV421 (BD Biosciences, San Diego, CA, USA), anti-CD38-PC7, anti-CD133-PE (EXBIO, Vestec, Czech Republic), anti-CD90-APC, and anti-CD45RA-FITC antibodies (Pharmigen, San Diego, CA, USA). The desired cell population was selected using a FACS Aria III cytometer (BD Biosciences, San Diego, CA, USA) (16).

#### Cell Culture

CD34^+^ or CD34^+^CD38^low^CD133^+^CD90^+^CD45RA^−^ cells were plated in Stem-alpha A medium without glucose (Stem Alpha SA, Saint-Genis-l'Argentiere, France), supplemented with penicillin/streptomycin (PS) (100 ng/L), and cytokines: SCF 100 ng/mL, IL-3 0.5 ng/mL, TPO 10 ng/mL. Cells were incubated under physiological conditions (3% O_2_, with glucose 1 g/L), anoxia (0% O_2_, with glucose 1 g/L), or anoxia/aglycemia (AA, 0% O_2_, without glucose) for 5–7 days at 37°C. The conditions with 3% O_2_ were obtained in an O_2_ and CO_2_ controller-culture chamber (PRO-OX and PRO-CO_2_, Biospherix, NY) (15). Anoxia was achieved using a hermetically sealed modular incubator chamber (Billups-Rothenberg, CA) in which ambient air was replaced with a mixture of 95% nitrogen and 5% CO_2_ (Air Liquide, Paris, France). At the end of the incubation period, cell expansion was estimated by cell counting.

### Apoptosis Assay

Apoptosis was detected with an Annexin V-FITC kit (Beckman Coulter, Carlsbad, CA, USA) according to the manufacturer's protocol. Briefly, 10^5^ cells from each of the experimental conditions were stained with Annexin V-FITC solution (AnnV) and propidium iodide (PI, 250 μg/mL) for 15 min at 4°C in the dark, washed in phosphate buffer saline (PBS), and analyzed with a flow cytometer (BD Bioscience, FACS Canto II) (17). This technique allow to detect: unlabelled viable cell subpopulation (AnnV^−^/ PI^−^); early apoptotic cell subpopulation that have bound only AnnV (Ann^+^/PI^−^); necrotic cell subpopulation (representing the cells in post-apoptosis necrosis or late apoptosis) that have both bound AnnV and have been labeled with PI (Ann^+^/PI^+^).

### Cell Cycle Analysis

For cell cycle analysis, 10^5^ cells were washed with PBS, centrifuged at 43 g for 5 min at 4°C, and the pellets were resuspended in 100 μl of PBS. Cells were then fixed with 900 μl of 70% cold ethanol (added dropwise), and incubated for 1 h at 4**°**C. Cells were washed and resuspended in PBS with 0.2 mg/mL of bovine RNase A and incubated for 1 h at 37°C in the dark. PI (10 μg/mL) was added, and the analysis was performed using flow cytometry (Bioscience, FACS Canto II) (17). The percentage of cells in each of the different cell cycle phases (G0/G1, S, and G2/M) was calculated from the linear graph on which cell counts were plotted relative to cell DNA content. The reagents were provided by Sigma Aldrich.

### CFC Assay

The committed hematopoietic progenitors', colony-forming cell (CFC), including colony-forming unit granulocyte, monocyte (CFU-GM), burst-forming unit erythroid (BFU-E), colony-forming unit granulocyte, erythrocyte, monocyte, megakaryocyte (CFU-GEMM) were assayed as described previously (18). Cells (250 cells/mL) were seeded in cytokine-supplemented methylcellulose in a 24-well-plate (MethoCult H4034 Optimum, Stem Cell Technologies, Canada). After 15 days of incubation, CFCs were counted using an inverted microscope.

### Aldehyde Dehydrogenase (ALDH) Activity

ALDH labeling was undertaken using an Aldefluor reagent (ALDF) (Stem Cell Technologies, Canada) according to the manufacturer's instructions. Activated ALDF substrate was rapidly added to a suspension of 10^5^ cells in 100 μL of ALDF assay buffer and incubated for 30 min at 37°C in the dark. As a negative control, an aliquot of ALDF-stained cells was incubated with the ALDH inhibitor diethylaminobenzaldehyde. Cells were washed, resuspended in 200 μL of assay buffer, and analyzed with a flow cytometer (BD Bioscience, FACS Canto II) (15).

### Proliferative, Clonogenic, and Differentiation Potential Evaluation by the Single-Cell Culture Method

The method was performed as it was previously published (16, 19).

#### Proliferative Capacity

Single cells selected from each of the experimental conditions were assayed into individual wells of 96-well-plates. Each well-contained 200 μl of the Stem alpha A medium, supplemented with glucose (1 g/l) and cytokines (see above). Plates were incubated at 20% O_2_ and 5% CO_2_ for 14 days at 37°C. The entire cell content produced in the primary culture from one individual cell was reseeded into 24-well-plates under the same culture conditions. Secondary culture cell content was re-evaluated at Day-28. Clone categories were established according to the number of cells produced: (C1 = 0; C2 = 1,000–4,000; C3 = 5,000–10,000; C4 = 13,500–14,000; C5 = 15,000–15,500 cells).

#### Clonogenic Capacity

Methylcellulose colony formation was assessed by sorting single cells from the different experimental conditions into individual wells of a 96-well-plate, containing 100 μl of methylcellulose (MethoCult H4034). The plates were incubated at 20% O_2_ and 5% CO_2_ for 15 days at 37°C. The colonies from the committed progenitors were then counted. Each colony was harvested, and replated into an individual well of a 24-well-plate containing methylcellulose (250 μl). The plates were incubated for 14 days at 37°C secondary colony formation was then assessed.

#### Differentiation Potential

For *in vitro* differentiation assays, cells from each of the experimental conditions were incubated for 14 days in the same manner as described for the proliferative capacity analysis. 24-well-plates had been seeded with murine stromal cells (MS5) in a αMEM medium (Gibco, Thermo Fisher, Langenselbold, Germany) with 10% fetal calf serum and PS (100 ng/L). At the end of the incubation period in liquid culture, hematopoietic cells were co-cultivated on MS5 for 30 days with a complete RPMI medium (Thermo Fisher, Langenselbold, Germany, with SCF 100 ng/mL, IL-3 0.5 ng/mL, G-CSF 100 ng/mL, TPO 10 ng/mL, 10% fetal calf serum, and PS) at 37°C. At the end of the incubation period, hematopoietic cells from each well were harvested and labeled at 4°C for 20 min in the dark with anti-CD19-PE, anti-CD33-APC (BD, Biosciences, San Diego, CA, USA), and anti-CD45-FITC antibodies (IOTest, Beckman Coulter, Carlsbad, CA, USA), and analyzed with a flow cytometer.

### Scid Repopulating Cells (SRC) Assay

All experiments involving animals were performed in compliance with French regulation (License No: 3306002). From each of the experimental conditions, the equivalent of 300 Day-0 CD34^+^CD38^low^CD133^+^CD90^+^CD45RA^−^ cells at Day-7 were injected into the retro-orbital vein of 6–10-week-old NOD-Shi-Scid/IL-2Rgnull (NOG) mice (animal-keeping facility, University of Bordeaux, France). The mice had been prepared using an intraperitoneal injection of Busulfan 25 mg/kg (Busilvex, Pierre Fabre, Boulogne, France) (20). Positive control mice (injected with 300 Day-0 CD34+CD38lowCD133+CD90+CD45RA- cells) and negative control mice (non-injected) were included. After 12 weeks, animals were euthanized and their femora were extracted. The femoral bone marrow was flushed with 1 ml of RPMI medium (Gibco, Thermo Fisher, Langenselbold, Germany) supplemented with 4% human serum albumin. The cells contained in 100 μl of this cell suspension were stained with anti-human antibodies: CD45-FITC, CD19-PE, and CD33-APC, for 20 min at 4°C in the dark, washed with PBS and analyzed on a FACS Canto II (21). The positivity threshold for human CD45, CD33, CD19 chimerism was >0.1% of analyzed cells.

### ATP Content

ATP levels were quantified using the ATP Bioluminescence Assay Kit HS II kit (Roche, Switzerland) in accordance with the manufacturer's recommendations, as it was previously published (15). A total of 10^3^ cells per well were used in a 96-well-plate. Luminescence, directly proportional to the concentration of ATP, was detected using a luminometer at α = 562 nm (Promega GloMax). In order to determine the total cellular and mitochondrial ATP production, cells were incubated for 10 min in the absence of or in the presence of inhibitors of the mitochondrial complexes I and III, rotenone, and antimycin (1 μM), respectively. These inhibitors were added simultaneously in order to curb electron flow through the mitochondrial electron transport chain, and so prevent generation of the proton motive force enabling ATP production by ATP synthase. The proportion of cellular ATP produced by mitochondria was calculated using the decrease in total ATP detected in the presence of these inhibitors.

### Mitochondrial Content

Mitochondria were detected using mitochondrial-selective fluorescent labeling with MitoTracker Green, according to the manufacturer's instructions (Molecular Probes). A total of 10^4^ cells were treated with Mitotracker green (100 nM) for 20 min at 37°C in the dark, then washed in PBS and analyzed using flow cytometry as previously published (17).

### Succinate Content

The succinate that had accumulated during cell culture was detected in the culture supernatant at the end of the incubation period according to the manufacturer's instructions (Succinate assay kit, Abcam, UK). Cell suspensions were harvested, washed and resuspended in 100 μl of ice-cold succinate assay buffer. The suspensions were centrifuged, and the collected supernatants were filtered using a 10 kDa spin filter. Fifty microliter of each sample or standard dilution were mixed with the same volume of the reaction mix or background control mix and incubated for 30 min at 37°C. The absorbance intensity was measured at 450 nm using a microplate reader (Evolis, Biorad). Each value was adjusted with the background control, in order to eliminate any non-specific absorbance that may have resulted from the possible presence of reduced nicotinamide adenine dinucleotide (NADH). Succinate concentration was then determined based on the standard curve.

### Statistical Analysis

The Wilcoxon-Mann–Whitney paired comparison test was used to examine the significance of the difference between the various experimental conditions (*p* < 0.05, *p* < 0.01, and *p* < 0.001 were considered to be statistically significant).

## Results

### Hematopoietic Stem and Progenitor Cell Survival in the Anoxia and AA

In order to test our hypothesis that primitive hematopoietic cells can survive under conditions commonly found in tumor tissue, we assayed both a total CD34^+^ population (CD34^+^) with a majority of hematopoietic progenitors (HPCs) and a rare minority of stem cells and a selected CD34^+^CD38^low^CD133^+^CD90^+^CD45RA^−^ population highly enriched in hematopoietic stem cells (HSCs, “stem”) under anoxic, anoxic/aglycemic (ischemia like, “AA”), or physiological conditions (3% O_2_) for seven days.

**Figure 1:**
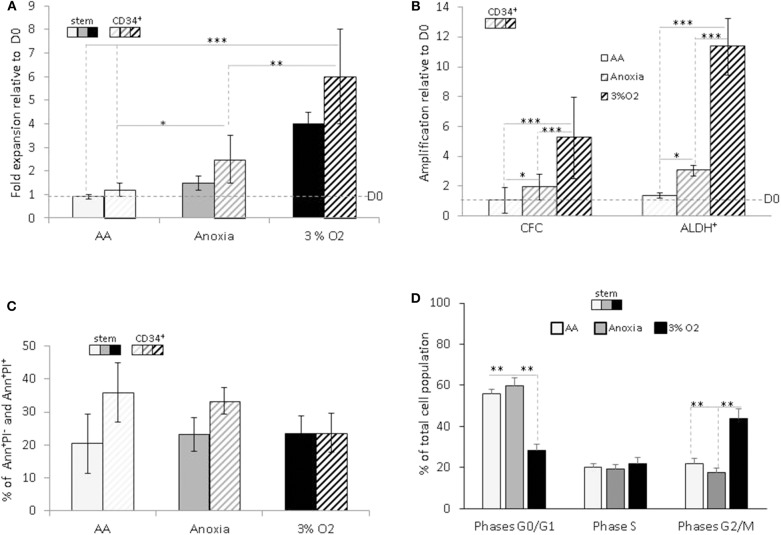
Hematopoietic stem and progenitor cell amplification, survival and cycling upon O_2_ and glucose shortage. The total CD34^+^ population (CD34^+^) and selected CD34^+^CD38^low^CD133^+^CD90^+^CD45RA^−^ highly enriched in hematopoietic stem cells (“stem”) population were analyzed after seven days of culture under anoxic/aglycemic (AA), anoxic, or physiological oxygen-level conditions (3% O_2_). **(A)** Evaluation of cell expansion. The bars represent mean fold expansion relative to Day-0 (D0). **(B)** Hematopoietic progenitor amplification. The average fold expansion of committed progenitors (CFC) or aldehyde dehydrogenase (ALDH^+^)-expressing primitive progenitors within the total CD34^+^ population relative to D0 is represented with bars. **(C)** Apoptosis assessment with Ann/PI labeling. The average percentage of apoptotic (Ann^+^/PI^−^) or necrotic cells (Ann^+/^PI^+^, i.e., cells in post-apoptosis necrosis or late apoptosis) detected amongst stem and CD34^+^ cells, is represented with bars. **(D)** Cell cycle analysis. Distribution of the cultivated “stem” cells in G0/G1, S, and G2/M phases of the cell cycle are represented by the average percentage shown with bars. The data are presented using a mean ±SD format over at least six experiments. Asterisks indicate a statistically significant difference between experimental conditions at *p < 0.05, **p < 0.01, or ***p < 0.001, Wilcoxon-Mann–Whitney test. Ann, Annexin; PI, Propidium iodide.

Based on the difference in cell numbers between Day-0 and post-incubation, the results showed that the entire cell population survived under AA conditions, in both the stem and CD34^+^ total population, with slight expansion in anoxia (Figure 1A). We decided to investigate whether this property was associated with the functional characteristics of hematopoietic cells within the heterogeneous CD34^+^ population. Primitive hematopoietic cells expressing ALDH, as well as committed progenitors incubated in anoxia, yielded a significant level of amplification compared to Day-0. The maintenance in number of both cell populations under AA conditions is indicative of metabolic adaptation to glucose and O_2_ shortage (Figure 1B). As expected, these conditions yielded a significantly lower number of cells than physiological conditions (3% O_2_), which allow optimal cell expansion (Figures 1A,B). Cellular viability was estimated using Annexin V/Propidium iodide labeling (Ann/PI). Similar percentage of apoptotic cells (including the subpopulation expressing Ann^+^/PI^−^) or necrotic cells (subpopulation expressing Ann^+^/PI^+^). was detected in both stem and CD34^+^ cells, under all conditions (Figure 1C). We further analyzed the cell cycle phase distribution with PI staining (Figure 1D). When incubated under restricted conditions, there was a significant accumulation of cells in the non-dividing G0/G1 phase of the cell cycle, compared to physiological conditions. However, a fraction (~40%) of the cell population was still actively proliferating (S and G2/M phases) in AA and anoxia (Figure 1D). This distribution could be responsible for the cultures' maintenance and/or slight expansion in the absence of O_2_ with/without glucose, allowing for a ~20% apoptosis rate. The observed survival of primitive hematopoietic cells, irrespective of the conditions (anoxia, AA) indicates their flexible metabolic nature.

### Stem Cell Functions Are Maintained *in vitro* in Anoxia and AA

The population enriched in stem cells was analyzed by single cell assay in order to detect stem cell traits—self-renewal, proliferation, and multipotency—*in vitro* at an individual cell level.

**Figure 2:**
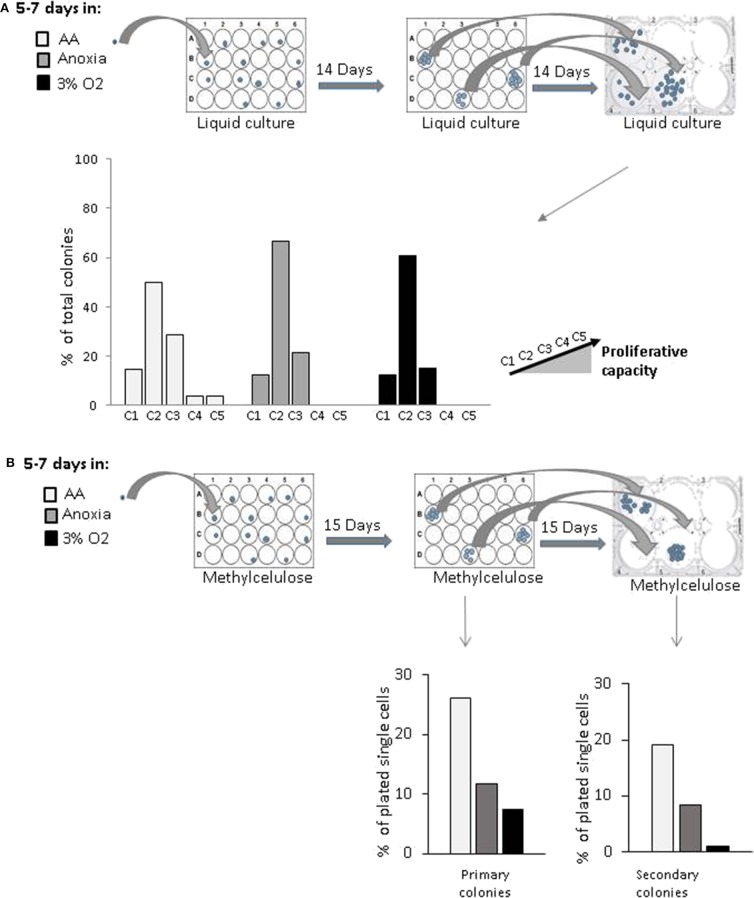
*in vitro* evaluation of proliferative and clonogenic potential of individual CD34^+^CD38^low^CD133^+^CD90^+^CD45RA^−^ cells in single-cell culture. CD34^+^CD38^low^CD133^+^CD90^+^CD45RA^−^ cells were incubated under anoxic/aglycemic (AA), anoxic, or physiological oxygen conditions (3% O_2_) for 5–7 days at 37°C and then analyzed. **(A)** Proliferation potential was assayed by selecting single cells from each experimental condition and placing these into individual wells. The cell progeny produced in primary culture from one individual cell were reseeded and secondary culture cell production was assessed. Clone categories were established according to the number of cells produced: (C1 = 0; C2 = 1,000–4,000; C3 = 5,000–10,000; C4 = 13,500–14,000; C5 = 15,000–15,500 cells). The data are shown with bars representing the percentage of total identified individual cell clones according to size **(B)** Clonogenic potential. Methylcellulose colony formation was assayed by sorting single cells from the various experimental conditions into individual wells. Primary colonies were then enumerated, replated individually in methylcellulose-containing wells and the secondary colonies scored. The results are displayed with bars representing the percentage of total identified individual cell cultures giving rise to primary colonies or secondary colonies.

In order to estimate the cell's proliferative capacity, we counted the number of cells produced in a secondary culture, having replated the cells descending from a single cell (seeded in a primary liquid culture) (Figure 2A). This secondary culture allowed us to detect both the total number of cells per well—total progeny of the initial single cell—and the proportion of clones produced by CD34^+^ cells in the primary culture. They were then sorted into five categories (see above, Material and methods). The more primitive the cell, the greater its progeny in the secondary culture, due to a higher proliferative potential. Our results showed that the cells producing the most proliferative clones (categories 4 and 5) were present only under AA conditions (Figure 2A). Using an approach based on methylcellulose culture, we found that the number of individual cells giving primary colonies (plating efficiency) was the highest under AA conditions (Figure 2B, Primary colonies). All cells from the individual cell-derived colonies (each primary culture well) were harvested, dissociated, and plated in individual wells in the secondary methylcellulose culture in order to assess replating efficiency (*in vitro* surrogate for “self-renewal”). Secondary colonies only grew from those primary colonies which contained self-renewed primitive progenitors. Replating efficiency was observed under all conditions, but the highest proportion of initially seeded single cells able to produce colonies in secondary culture was detected under AA conditions (Figure 2B, Secondary colonies). We then tested if these primary colonies could undergo myeloid or lymphoid lineage differentiation on a mesenchymal stroma layer, proving their multipotency. For this, we ascertained the percentage of human CD45^+^ cells (a pan-hematopoietic cell marker) expressing markers of the myeloid (CD33^+^) or lymphoid (CD19^+^) lineage differentiation. The proportions detected were equal under all culture conditions (Supplementary Figure 1). The results indicate that the basic stem cell functions of self-renewal and multipotency are not comprised by incubation in anoxia or AA.

### Long-Term *in vivo* Hematopoietic Reconstitution Upon Transplantation of the Cells Cultivated in Anoxia and AA

**Figure 3:**
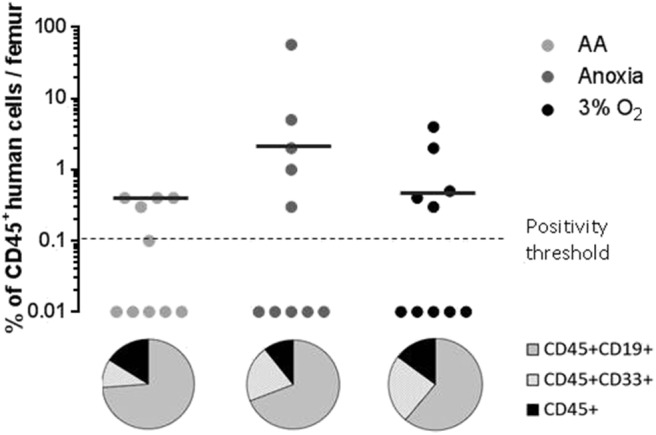
Long-term hematopoietic reconstitution capacity of the CD34^+^CD38^low^CD133^+^CD90^+^CD45RA^−^ cultured in the absence of O_2_ and with/without glucose. Scid repopulating activity of CD34^+^CD38^low^CD133^+^CD90^+^CD45RA^−^ cells cultivated in AA, anoxia or 3% O_2_ was evaluated based on human CD45^+^ chimerism in the bone marrow of NOG mice, 12 weeks after injected cultures with the Day-7 equivalent of 300 Day-0 cells. Femoral bone marrow cells were labeled with human CD45 antibodies and the level of human CD45 chimerism was assimilated to the amount of CD45^+^ cells in the mouse bone marrow. The data are displayed using circles, with each mouse being represented by one circle. Median values of CD45 chimerism are indicated by dashed lines (upper panel). Human myeloid and lymphoid cell differentiation was estimated with the level of expression of human CD45^+^CD33^+^ and CD45^+^CD19^+^ markers, respectively, in mouse bone marrow. The average percentage of human CD19 and CD33 expression in the human CD45^+^ population are presented in the pie chart (lower panel).

When assaying stem cells, the most effective method is to track their ability to reconstitute haematopoiesis in immunosuppressed mice 12–16 weeks after transplantation (22, 23). We analyzed the presence of the human cell marker CD45 12 weeks after xeno-transplantation of NOG mice. We found significant human cell engraftment in the mice's bone marrow (Figure 3, upper panel). Graft efficiency was defined as the number of mice testing positive for human CD45^+^ cells relative to the total number of mice injected. This ratio was equal for all experimental conditions (Figure 3, upper panel). The mean percentage of CD45^+^ cells—level of CD45 chimerism—was consistent in AA and anoxia as well as under physiological conditions. These results indicate a long-term ability maintained by the stem cell population, throughout incubation, for hematopoietic reconstruction, a trait attributable to the most primitive HSCs. CD45^+^CD33^+^ and CD45^+^CD19^+^ markers were then used to detect human myeloid or lymphoid differentiation, respectively (Figure 3, pie chart). This property was again detected under all conditions. The ratio of CD45^+^CD33^+^ to CD45^+^CD19^+^ cell subpopulations indicated that cells incubated in AA tend toward lymphoid lineage more than in anoxia and 3% O_2_ (Figure 3, pie chart).

### ATP Production, Active Mitochondrial, and Succinate Content in Hematopoietic Stem and Progenitor Cells in Anoxia and AA

**Figure 4:**
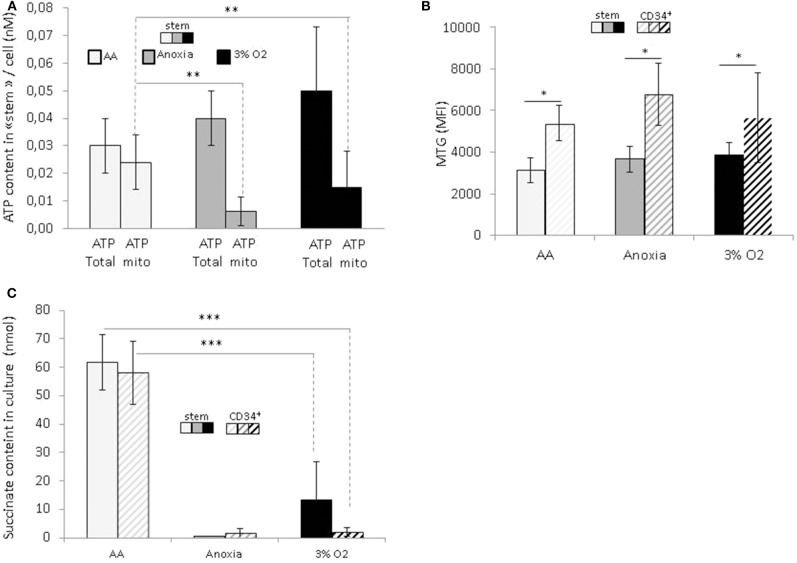
Energetic profile of hematopoietic stem and progenitor cells and succinate accumulation in culture upon O_2_ or O_2_ and glucose shortage. At the end of the 7-day incubation period, total CD34^+^ cells and stem cells (CD34^+^CD38^low^CD133^+^CD90^+^CD45RA^−^) were analyzed **(A)** ATP level assessment. The bars represent the mean cellular total or mitochondrial ATP level. The data are presented as a mean ± SD from five experiments **(B)** Mitochondrial mass is determined by MTG staining and the results are presented as the MTG mean fluorescence intensity (MFI). The data are displayed as the mean ± SD of four independent experiments. **(C)** Succinate content determination in the culture supernatant. Data are presented as the mean ± SD of three independent measurements. Asterisks indicate a significant difference between the different experimental conditions at *p < 0.05, **p < 0.01, or ***p < 0.001, Wilcoxon-Mann–Whitney test.

In order to reveal the specific bioenergetic profile enabling hematopoietic cell survival without O_2_ or glucose, we analyzed the cellular ATP content (Figure 4A). Firstly, this showed a significant decrease in the total level of ATP in anoxia and AA compared to 3% O_2_, indicating a decrease in cellular ATP production. After simultaneous introduction of inhibitors of mitochondrial complexes I and III (rotenone and antimycin, respectively), the decrease in total ATP indicated the fraction of ATP produced by mitochondria. We found that most of the ATP in AA is produced by mitochondria; apart from the observation that anaerobic mitochondrial ATP production is active in AA, the simultaneous addition of inhibitors prevented us from distinguishing the specific effect of either complex (Figure 4A). This phenomenon was observed in both the stem and total CD34^+^ cell populations. To see whether these changes were associated with a difference in mitochondrial mass content, we labeled the cells with Mitotracker green at the end of the incubation period. This is a compound which binds to active polarized mitochondria, indicating cellular mitochondrial mass (24). We found a similar quantity of active mitochondria per cell under all conditions, amongst stem cells as well as the total CD34^+^ population (Figure 4B). This would suggest that mitochondrial content was not affected by the absence of O_2_ or glucose. Furthermore, compared to HSCs, the mitochondrial content in CD34^+^ cells was significantly higher under all conditions, indicating that in terms of bioenergetics, progenitor cells are more dependent on mitochondrial respiration than stem cells (Figure 4B).

In a recent publication, Tomitsuka et al. described how cancer cells can survive in ischemia where anaerobic mitochondrial fumarate respiration is activated (25). Using this fumarate as the final electron acceptor, then it is reduced into succinate that accumulates during culture. To test if, under our AA conditions, anaerobic mitochondrial respiration works in the same manner as in cancer cells, we measured the level of succinate in culture supernatants. The results show that the succinate content in AA supernatant is significantly higher compared to 3% O_2_ and anoxia (Figure 4C). These results point clearly to succinate accumulation under ischemia-like conditions and suggest the activation of fumarate respiration as an energetic pathway in HSCs/HPCs under these circumstances. Interestingly, this was detected in stem and CD34^+^ cells, indicating that HSCs as well HPCs can exhibit metabolic adaptions to survive in ischemia-like conditions and activate anaerobic mitochondrial respiration.

## Discussion

Our study demonstrates that HSCs/HPCs are capable of maintaining their functional traits, high proliferative capacity, and multipotency in the absence of O_2_ and/or glucose. Although cell numbers were significantly reduced in comparison with physiological conditions, a significant fraction of surviving cells was actively cycling independent of O_2_ and/or glucose omission (Figure 1). Moreover, primitive CB progenitors and stem cells (ALDH^+^), as well as committed progenitors, were even seen to multiply despite the absence of O_2_, indicating an oxygen-independent mechanism, which is arrested by glucose shortage. This is consistent with data showing a link between glucose import and cell proliferation in normal as well as cancer cells, in order to provide the carbon intermediates for biosynthesis (4, 26).

Using our method, the greatest capacity to adapt was detected in the stem cell population, wherein SRC (Scid repopulating cell) activity was equivalent to that which was detected under optimal physiological conditions (Figure 3). Notably, under ischemia-like conditions, we identified the cell subpopulation with the highest proliferative capacity, indicating an association between high metabolic robustness and the most primitive of cells.

Various articles describe how O_2_ is a functional regulator of HSCs/HPCs. Our results are consistent with and could indeed extend, those showing that although low O_2_ concentration, close to that found in bone marrow stem cell niches (0.1%), induced the return of half of the CB CD34^+^ cells or murine hematopoietic progenitor non-leukemic factor-depending cell Paternsen (FDCP) mix-cell line to G0 phase, it also selected the small proportion of self-renewing cells in the proliferating phase of cycle, without impairing their functional capacity, SRC activity, or clonogenicity (27, 28). Further, a 1% O_2_ concentration enables HSC enrichment as well as primitive erythroid progenitors within CB CD34^+^ cell population (29–31). In addition, our study confirmed that well-adapted physiologically-relevant low O_2_ concentration (3%) allows simultaneous HSC maintenance and committed progenitor expansion (18).

These data reflect the behavior of HSCs/HPCs in a physiological environment. Adult or neonatal primitive hematopoietic cells reside in low oxygen areas and develop within a 1–4% gradient of O_2_ (32, 33). Furthermore, as has been shown, most primitive HSCs reside in areas with very low blood perfusion, implying a limited supply not only of O_2_ but also of blood-born nutrients (34), i.e., an ischemia-like environment is common to HSCs. In the niche, HSCs are in a quiescent phase, and are occasionally recruited into the cell cycle (35, 36). They must also remain capacity for rapid proliferation in response to extrinsic cues, indicating great metabolic plasticity which enables energetic reprogramming on the basis of various functional demands (2).

These properties are in common to cancer cells. While cell growth is markedly reduced under conditions of very low O_2_ levels, cancer cells (leukemic cell culture) are capable of expanding significantly compared to Day-0 (37). These hypoxia-resistant cells shown to be highly immature progenitors (37). Also, severe hypoxia selects *ex vivo* a cell subset with stem cell potential in a population of myelodysplastic syndrome bone marrow cells (38). The cells that survive under these conditions have been shown to be the leukemic stem cells which are potentially responsible for the disease relapsing (39). We have demonstrated here for CB HSCs/HPCs, as has been shown for cancer cells, that expansion is possible at a low oxygen level until glucose is no longer available in the culture medium. In summary, these data indicate that primitive hematopoietic cells, as well as cancer progenitor and stem cells, adapt to survive anoxia and ischemia, conditions often found in cancerous tissue.

Furthermore, our results show that under ischemia-like conditions, primitive hematopoietic cell mitochondria remain functional, and use anaerobic ATP synthesis in order to sustain cellular homeostasis (Figure 4). Recently, evidence has shown fumarate respiration to be an anaerobic energetic pathway utilized by cancer cells in ischemic tumor tissue. In this instance, fumarate is utilized as the final electron acceptor, and fumarate reduction is coupled with an anaerobically-functioning electron transport chain in which electrons are transferred from NADH to fumarate via the complex I, ubiquinone, and a reverse reaction of the complex II (fumarate reductase), resulting in succinate formation. Only complex I functions as a proton pump, generating a proton gradient that drives ATP synthesis (25).

The accumulation of succinate we detected suggests that primitive hematopoietic cells activate fumarate respiration under ischemia-like conditions (Figure 4), meaning that HSCs/HPCs could in fact adapt by using the same metabolic energetic solvating pathway as cancer cells. By introducing a fumarate reductase-specific inhibitor, and assessing HSC/HPC population maintenance, we were able to identify activation of the NADH fumarate reductase system. We observed that introducing pyrvinium pamoate (which inhibits NADH fumarate reductase), prevents mesenchymal stem cells surviving under ischemia-like conditions, and triggers energetic failure (unpublished data, article in preparation). Based on these findings and the results presented in this article, we propose that this system could be activated under ischemia-like conditions in HSCs/HPCs.

It should be noted that when analyzing the metabolic pathway in anaerobic respiration, such analysis should include fatty acid β-oxidation (a known alternative energetic fuel in the face of a glucose shortage) as fatty acid β-oxidation yields reducing equivalents of nicotinamide adenine dinucleotide (NADH) (40). These could deliver electrons to the electron transport chain (complex I), thereby sustaining anaerobic mitochondrial ATP generation via fumarate respiration in an anoxic and aglycemic environment.

Our study provides evidence that primitive hematopoietic cells exhibit metabolic and energetic flexibility, notably the ability to shift between aerobic, or anaerobic energetic modes in order to adapt to various environmental conditions. These features, especially “fumarate respiration,” were thus far considered to be specific to cancer cells, which is clearly not the case. We propose that since they are common to both, normal and cancer stem cells, these primitive metabolic features should be considered as markers of stemness.

Our work provides findings that could be important in the development of cell therapy procedures.

## Data Availability Statement

All datasets generated for this study are included in the article/Supplementary Material.

## Author Contributions

VL, AB, AR, PD, and LR performed the experiments. PB provided intellectual input and helped with data analysis. NG edited the text and corrected the translation. ZI provided the original idea for the project. MV-L and ZI conceived the study and wrote the manuscript. MV-L supervised the experimental work and did the data analysis.

## Conflict of Interest

The authors declare that the research was conducted in the absence of any commercial or financial relationships that could be construed as a potential conflict of interest.

## Supplementary Material

Supplementary Figure 1 *in vitro* evaluation of the differentiation potential of individual CD34^+^CD38^low^CD133^+^CD90^+^CD45RA^−^ cells in single-cell culture. Single cells were selected from the various experimental conditions (AA, anoxia, or 3% O_2_), and plated into individual wells. Clones produced in primary culture from one individual cell were co-cultivated on a mesenchymal stromal layer for 30 days. Cells were then collected and assayed for myeloid (CD45^+^CD33^+^) or lymphoid differentiation (CD45^+^CD19^+^). Results are shown with bars representing the mean percentage of CD45^+^CD33^+^ or CD45^+^CD19^+^-expressing cells obtained per colony developed from one individual cell.

## Abbreviations

AA: anoxia/aglycemia
ALDH: aldehyde dehydrogenase
Ann/PI: annexin/propidum iodide
ATP: adenosine triphosphate
CB: cord blood
CFC: colony forming cell
CSCs: cancer stem cells
IL-3: interleukin-3
HPCs: hematopoietic progenitor cells
HSCs: hematopoetic stem cells
MFI: Mean fluorescence intensity
MTG: Mitotracker green
NAD: nicotinamide adenine dinucleotide
SCF: stem cell factor
TCA: cycle-tricarboxylic acid cycle
TPO: thrombopoietin.
