# Supplementary figures and images for: Normal Hematopoetic Stem and Progenitor Cells Can Exhibit Metabolic Flexibility Similar to Cancer Cells
Source: Front Oncol. 2020 May 12;10:713. doi: 10.3389/fonc.2020.00713 (PMC7247845; doi:10.3389/fonc.2020.00713)

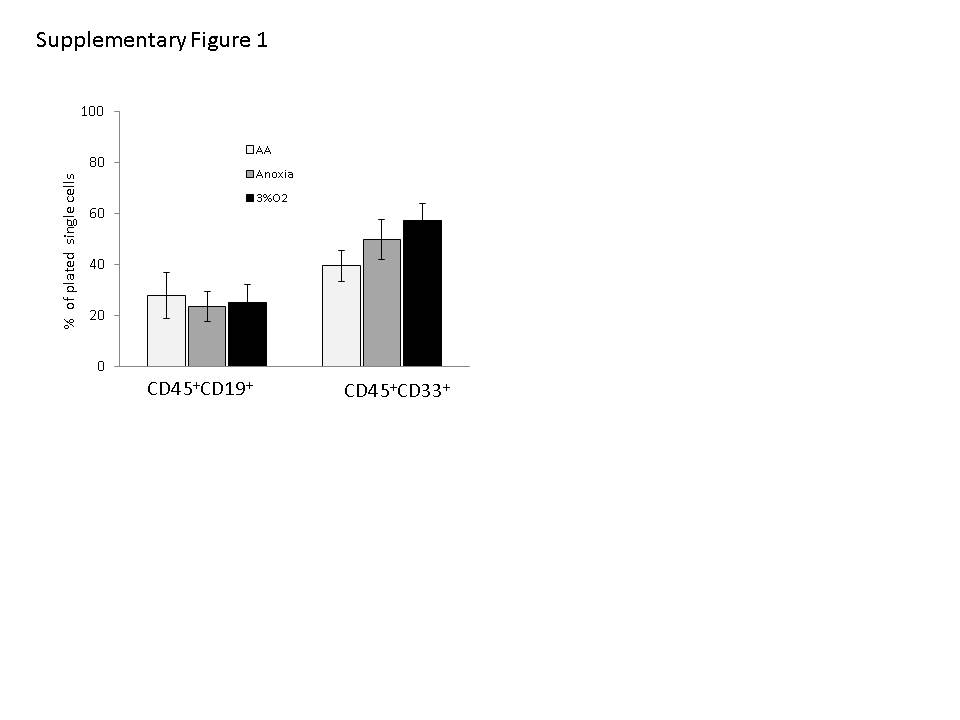

Supplement: Supplementary Figure 1 — in vitro evaluation of the differentiation potential of individual CD34+CD38lowCD133+CD90+CD45RA− cells in single-cell culture. Single cells were selected from the various experimental conditions (AA, anoxia, or 3% O2), and plated into individual wells. Clones produced in primary culture from one individual cell were co-cultivated on a mesenchymal stromal layer for 30 days. Cells were then collected and assayed for myeloid (CD45+CD33+) or lymphoid differentiation (CD45+CD19+). Results are shown with bars representing the mean percentage of CD45+CD33+ or CD45+CD19+-expressing cells obtained per colony developed from one individual cell. [file Image_1.JPEG]
